# Pilot study in Hamburg on the prevalence of SARS-CoV-2 infections and pandemic survey in the German funeral industry

**DOI:** 10.1007/s12024-023-00661-y

**Published:** 2023-06-24

**Authors:** Julia Schädler, Marc Lütgehetmann, Ann Sophie Schröder, Carolin Edler, Klaus Püschel, Benjamin Ondruschka, Antonia Fitzek

**Affiliations:** 1 Institute of Legal Medicine, University Medical Center Hamburg-Eppendorf, Hamburg, Germany; 2 Institute of Medical Microbiology, Virology, and Hygiene, University Medical Center, Hamburg-Eppendorf, Hamburg, Germany

**Keywords:** Funeral and crematory workers, SARS-CoV-2, Vaccination, COVID-19, Seroprevalence, Occupational health and safety

## Abstract

Funeral home and crematorium workers are an important occupational group in the corona crisis. The occupational setting led to concerns about an increased risk of infection with SARS-CoV-2. The seroprevalence in this occupational group is unclear. A questionnaire-based retrospective survey of funeral home and crematorium staff was conducted in December 2020. A second survey of funeral and crematorium staff was conducted 6 months later, in June 2021, to determine changes in pandemic management. Seroprevalence or vaccination status for SARS-CoV-2 was determined at these two time points. In December 2020, a seroprevalence of 2.3% (*n* = 1/44) was detected in funeral home and crematorium workers. In June 2021, one additional participant tested positive for the SARS-CoV-2 nucleocapsid. Of the participants, 48.5% (*n* = 16) were vaccinated at this time. The risk of SARS-CoV-2 infection for funeral home and crematorium workers is more similar to that of the general population in Hamburg, Germany. We found no evidence of an increased risk of infection at these two time points in our cohort. Further education on communicable diseases or appropriate protective measures in this occupational group for other infectious diseases would be useful in the future.

## Introduction

During the corona crisis, the increased mortality rate led to an increase on fatalities and funerals, making funeral and crematory workers an essential profession on every day of the coronavirus disease 2019 (COVID-19) pandemic. The pandemic has featured the significance of burial service labors as an essential organization of a frequently overlooked on-call specialization during public and worldwide emergencies. However, little is yet known about the risk of infection from the management and handling of deceased COVID-19 patients and their hygiene concepts during the pandemic period [1]. This applies not only to medical professions, but also to often forgotten groups of people such as funeral, crematorium, and church staff, as well as relatives of the deceased.

It has already been generally shown that funeral and crematorium workers have a certain risk of contracting an infectious disease in the course of their professional activities, as they often do not know the exact cause of death of a person (e.g., due to professional confidentiality) [2–4]. Due to the initial lack of information on the contagiousness of deceased severe acute respiratory syndrome corona virus 2 (SARS-CoV-2) victims, initial recommendations discouraged autopsies, ritual washings, or enactments [5–9].

In the course of previous pandemics, the Institute of Forensic Medicine Hamburg (ILM), as well as undertakers, crematorium staff, and church employees, has received many enquiries from relatives who wanted information about the course of the disease and the ban on contact beyond death until burial.

At the time of the study, several recommendations for dealing with SARS-CoV-2 deceased persons and a prioritization of vaccination for certain vulnerable occupational and population groups in Germany had been added [5, 6, 10]. Employees of the funeral trade were not included, however, as they were not previously considered medically relevant [11].

The aim of this study was to investigate the seroprevalence of SARS-CoV-2 among employees of funeral homes and crematoria in Hamburg during and after the second pandemic wave in Germany and to investigate the relevance and effectiveness of infection control measures in dealing with SARS-CoV-2-associated deaths in funeral homes and crematoria.

## Methods

### Study design—questionnaire

At ILM Hamburg, a self-designed questionnaire was distributed to employees of funeral homes and crematoria in Hamburg who were professionally directly involved with persons in Hamburg who had died from SARS-CoV-2.

Participation was voluntary, anonymous, and not remunerated financially or otherwise. At the ILM, the questionnaires were available in an area accessible to employees of funeral homes, consecutively numbered and with a predefined study ID. The number of questionnaires was not limited. The study ID numbers were continuously generated. The completed questionnaires were dropped into a designated box in the ILM by the participants.

The first survey period covered the first 2 weeks in December 2020 (part 1). After 6 months, in June, a second anonymous survey was conducted with a questionnaire aimed at capturing possible changes in their professional environment during the pandemic (part 2).

The second questionnaire was available at the ILM as described above. Participants were asked in the questionnaire if they remembered their study ID number from the first survey, and if so, they provided it in the second survey.

The questionnaires in December 2020 and June 2021 each contained a brief explanation of the study objectives and asked participants about their previous experience with the corona pandemic. The questionnaire consisted of ten and seven questions respectively (see electronic supplementary material).

### Testing for past SARS-CoV-2 infection and vaccination

Employees of funeral homes and crematoria were additionally informed in the questionnaires that they could voluntarily provide a blood sample to determine their SARS-CoV-2 antibody status for infection and vaccination. Therefore, each participant could voluntarily provide their study ID at both time points of blood sample collection allowing for comparison of the respective samples.

Employees of funeral homes and crematoria were screened for seroprevalence of SARS-CoV-2 infection in their blood at two time points, in December 2020 and June 2021. These analyses were carried out at the Institute for Microbiology and Virology of the UKE. The presence of previous infections was determined by SARS-CoV-2 antibodies (IgG/IgM/IgA) against the viral nucleocapsid using the Cobas e411 system (Roche Diagnostics, Manheim, Germany). To confirm vaccination status, we performed quantitative detection of viral spike protein at the second intervention time point in June 2021. SARS-CoV-2 S (RBD) antibodies (IgG/IgM/IgA; Cobas e411 system) and the SARS-CoV-2 S trimer assay (Liaison XL, DiaSorin Saluggia, Italy) were used. All assays were performed according to the manufacturer’s recommendations.

The cut-off for antibody reactivity against SARS-CoV-2 spike RBD is 0.8, for SARS-CoV-2 spike-trimer IgG 32 BAU/ml, and/or SARS-CoV-2 nucleocapsid 1 BAU/ml.

The evaluation of the results was anonymous and served exclusively for scientific purposes. The blood samples were discarded afterwards. Each subject signed a written informed consent form prior to blood collection. Participation in the December blood collection was not a prerequisite for participation in June.

### Statistical analysis

Statistical analysis was performed descriptively using Microsoft Excel (version 16.16, Microsoft Corporation, Redmond, USA). Variables were described as percentages, means, and standard deviations (SD). The assumption of a normal distribution was checked. Categorical variables were compared using the chi-square test. Graphical representation of results was performed using GraphPad Prism® statistical software (version 8.0, GraphPad Software Inc., La Jolla, USA).

## Results

### Employees of funeral homes and crematoria

#### Part 1: December 2020

##### General information

Forty-four questionnaires were distributed and answered. Of the participants, 20.5% (*n* = 9) were female and 79.5% (*n* = 35) were male. The mean age was 42.7 (SD ± 12.51) years (see Table 1).

**Table 1:** Main aspects of the results of the questionnaire for funeral and crematory workers—Part 1: December 2020

				**Participants** (*n* = 44)
General information				
Age, years			Mean (SD)	42.7 (12.51)
Gender			Male	35 (79.5%)
			Female	9 (20.5%)
Hygienic protective measures				
No briefing				3 (6.8%)
Single entry				10 (22.7%)
Multiple choice				31 (70.5%)
Normal handling of the decedents with face masks* for the employees^a^				20 (45.5%)
Normal handling of the decedents with face masks* for the employees and medical face mask for the decedents^a^				8 (18.2%)
Face masks* for the employees and orienting view^a^				8 (18.2%)
Face masks* for the employees and no opening of the body bag^a^				23 (52.3%)
Transport in the coffin without contact with the corpse^a^				9 (20.5%)
Other protective measures (disposable coat, gloves, glasses)^a^				25 (56.8%)
Other^a^				2 (4.6%)
Compliance with the hygienic protective measures				
None				1 (2.3%)
Partially adherence				10 (22.7%)
Full adherence				30 (68.2%)
No answer				3 (6.8%)
Complaints^b^ of relatives				
Yes				20 (45.5%)
No				15 (34.0%)
No answer				9 (20.5%)

This table shows general information about the participants and the hygiene measures taken and their compliance until December 2020. *Face masks include surgical face masks, FFP2 masks, or FFP3 masks; ^a^multiple choice was possible; ^b^complaints regarding no existing farewell ceremonies at the crematory during the pandemic

##### Hygienic protective measures

Of the participants, 6.8% (*n* = 3) were not informed by their employer on how to deal with people who died of SARS-CoV-2. The recommendations for protective measures given to workers by their companies are shown in Table 1. Of the participants, 68.2% (*n* = 30) adhered to the recommendations given by their workers; 2.3% (*n* = 1) reported not complying with the protective measures; and 22.7% (*n* = 10) partially complied. Reasons given were a lack of labeling of the coffins, lack of information about possible infections, or the sealing of the coffins. Individual factors such as fogging of glasses and forgetting protective measures in stressful situations were also mentioned. 6.8% (*n* = 3) did not answer this question.

##### Subjective perceptions

Overall, 59.1% (*n* = 26) felt only partially protected or not protected at all and 38.6% (*n* = 17) felt inadequately informed about how to deal with (possible) SARS-CoV-2 deaths (see Table 2).

**Table 2:** Subjective perceptions of the participants during the pandemic

		**December 2020** (*n* = 44)	**June 2021** (*n* = 33)	***P*-value**
Protection of infection				
Felt sufficiently protected		17 (38.6%)	16 (48.5%)	0.257
Felt partially protected		23 (52.3%)	12 (36.4%)	
Felt unprotected		3 (6.8%)	5 (15.2%)	
No answer		1 (2.3%)	-	
Information				
Well informed		26 (59.1%)	22 (66.7%)	0.499
Inadequately informed		17 (38.6%)	12 (36.4%)	
Indecisive		0 (0.0%)	1 (3.1%)	
No answer		1 (2.3%)	-	

This table shows the different subjective perceptions of the participants regarding the risk perceived and information of occupational exposure when handling SARS-CoV-2-infected deceased comparing December 2020 survey with June 2021 survey. *P* values as a result from chi-square tests

##### Complaint management

Of the participants, 45.5% (*n* = 20) were sometimes informed of complaints from family members due to a missed farewell/funeral service or cremation (see Table 1).

##### SARS-CoV-2 infection

Knowingly, none of the participants had reported a confirmed SARS-CoV-2 infection.

Rapid or qPCR testing in the occupational setting (March 2020 to December 2020).

None of the participants had been tested positive for SARS-CoV-2 in the past in the occupational setting by rapid antigen tests or nasopharyngeal swabs. In no case did the employer perform intermittent or periodic testing for SARS-CoV-2 infection (see Table 3). However, 59.1% (*n* = 26) would have liked to do so to increase their sense of safety at work. 15.9% (*n* = 7) did not think this was necessary, and 25.0% (*n* = 11) did not know about the existence of rapid antigen testing.

**Table 3:** Infection and seroprevalence for SARS-CoV-2

			**December 2020** (*n* = 44)	**June 2021** (*n* = 33)
			0 (0.0%)	1 (3.0%)
SARS-CoV-2 testing initiated by the employer				
No			44 (100.0%)	17 (51.5%)
Yes			0 (0.0%)	16 (48.5%)
Positive antibody reactivity			1 (2.3%)	18 (54.5%)
Infection-specific			1 (2.3%)	2 (6.1%)
Vaccination-specific			0 (0.0%)	16 (48.5%)
Vaccination status			0 (0.0%)	16 (48.5%)
One vaccination			0 (0.0%)	3 (9.1%)
Two vaccinations			0 (0.0%)	13 (39.4%)
Received vaccination				
Comirnaty® vaccine (BioNTech/Pfizer)				11 (68.8%)
Spikevax® vaccine (vaccine Moderna)				4 (25.0%)
Vaxzevria® vaccine (AstraZeneca)				1 (6.2%)

This table shows SARS-CoV-2 testing by employer, positive antibody reactivity, vaccination status of workers, and vaccination received in December 2020 and June 2021

##### Suggestions for improvement for a next pandemic

Suggestions for improvement included regular virus testing and equal staffing/teams, e.g. in one vehicle/shift. More comprehensive information about infection routes, pathogens, and protective measures was also desired. Other points of criticism mentioned were inconsistent, unsystematic regulations between individual units of a company and structural deficiencies (blowers aimed directly at coffins). Several persons wished more external control measures as part of the protection concept.

#### Part 2: June 2021

##### General information

Thirty-three questionnaires were distributed and answered. 15.2% (*n* = 5) were female, and 84.8% (*n* = 28) were male. The mean age was 42.85 (SD ± 13.56) years.

##### SARS-CoV-2 infection

Of the participants, 12.1% (*n* = 4) reported experiencing subjective symptoms of COVID-19-like illness such as cough, fever, loss of taste, or general cold symptoms in the last 6 months. One of them had a PCR-confirmed SARS-CoV-2 infection (*n* = 1; 3.0%). The infection occurred in a private setting (see Table 3).

##### COVID-19 vaccination

A total of 48.5% (*n* = 16) were partially or fully vaccinated at the time of the second survey. The distribution of the vaccine used and the number of vaccinations in detail are shown in Table 3.

##### Hygienic protective measures

The distribution of changes in hygiene protection measures compared to December 2020 is shown in Table 4. From April 2021, some participants were offered a regular SARS-CoV-2 rapid test, but this was not mandatory.

**Table 4:** Changes in hygienic protective measures in June 2021

			**Participants** (*n* = 33)
Choices			
No briefing			1 (3.0%)
Single entry			16 (48.5%)
Multiple choice			16 (48.5%)
Changes compared to December 2020			
Same			16 (48.5%)
Tightened			8 (24.4%)
Loosed			1 (3.0%)
Regularly controlled			5 (15.2%)
Subjectively improved			3 (9.1%)
SARS-CoV-2 rapid antigen testing offered			15 (45.5%)

This table shows the changes in hygienic protective measures in June 2021 compared to December 2020

##### Subjective perceptions

Of the participants, 51.5% (*n* = 17) felt partially or insufficiently protected in the professional environment. Another 36.4% (*n* = 12) felt inadequately informed about how to deal with (potential) SARS-CoV-2-deceased persons (see Table 2).

##### Estimated number of contacts with SARS-CoV-2-infected deceased

Of the participants, 81.8% (*n* = 27) of the participants reported estimated contacts with 5 to 1000 SARS-CoV-2-infected deceased persons (median 250; range: 0–1000). A participant may have come into contact with a corpse more than once, e.g., if they transported it at two different times (outward and return transport).

### Seroprevalence for SARS-CoV-2 in the funeral industry

#### Part 1: December 2020

Blood samples were collected from 44 external ILM staff, including funeral home and crematorium staff, to detect a possible positive serological reaction to SARS-CoV-2 antibodies. SARS-CoV-2 antibodies to the nucleocapsid, indicating previous infection, were detected in only one case (2.3%).

#### Part 2: June 2021

**Fig. 1:**
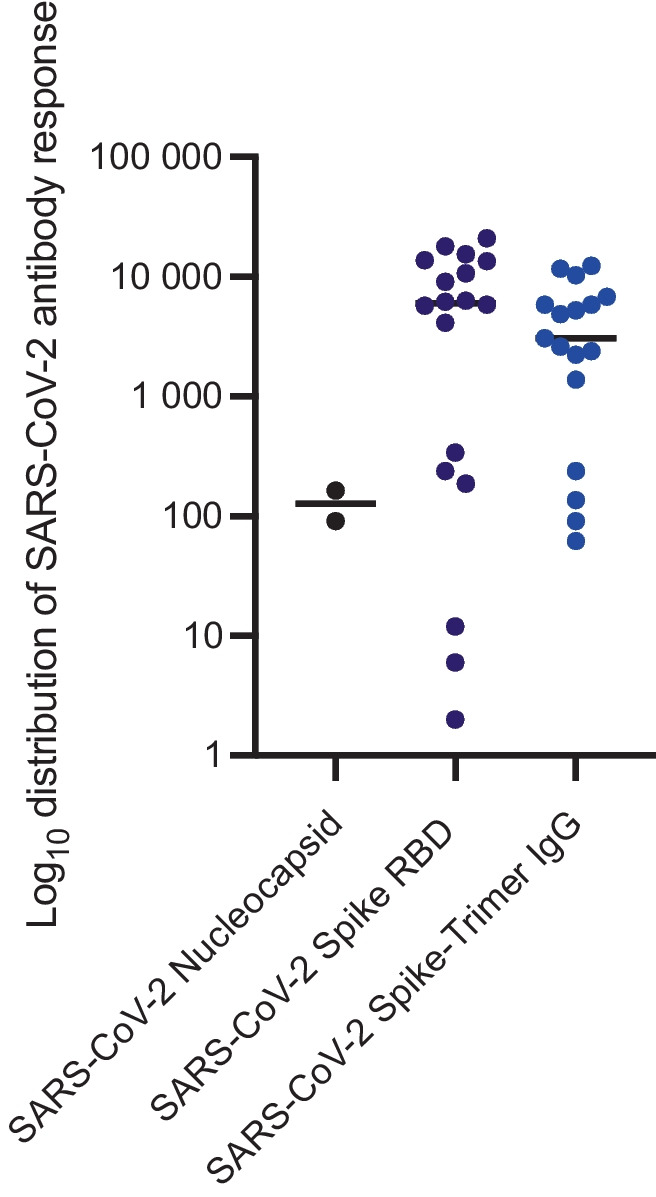
Distribution of SARS-CoV-2 antibody response. This figure shows the antibody reactivity against SARS-CoV-2 spike RBD (dark blue, cut-off: 0.8), SARS-CoV-2 spike-trimer IgG (light blue, cut-off: 32 BAU/ml), and SARS-CoV-2 nucleocapsid (black, cut-off: 1 BAU/ml) in 18 participants in June 2021. The results of the distribution of SARS-CoV-2 antibody response were shown logarithmized

Thirty-three blood samples were collected using the same procedure as in December 2020. Twenty-seven of them participated at both time points. Fifteen participants had not developed antibodies in June 2021. Eighteen participants showed positive antibody reactivity against SARS-CoV-2 spike RBD (cut-off: 0.8), SARS-CoV-2 spike-trimer IgG (cut-off: 32 BAU/ml), and/or SARS-CoV-2 nucleocapsid (cut-off: 1 BAU/ml). Two of these participants (6.1%) developed antibodies to all the SARS-CoV-2 virus structures tested, corresponding to a past infection (see Fig. 1). Presumably, one of them was the already known antibody-positive participant from December 2020, and the other person had a known PCR-confirmed SARS-CoV-2 infection between December 2020 and June 2021. In addition, 15 participants reacted positively to SARS-CoV-2 spike RBD and SARS-CoV-2 spike-trimer IgG. Another person reacted positively only to SARS-CoV-2 spike RBD. In total, 16 people were vaccinated.

## Discussion

For funeral and crematorium workers, many questions about the handling and infectivity of deceased persons with questionable or proven SARS-CoV-2 infection remained unanswered, leading to a constant fear of infection in the professional environment.

This is the first study known to date to investigate the potential risk of infection by determining the prevalence of SARS-CoV-2 infection among funeral home employees in Hamburg at two time points 6 months apart. The attached questionnaires underline that funeral professional in Germany still belongs to a “forgotten professional environment” in terms of handling infectious deceased persons.

In our study cohort, only one person had become infected by December 2020 (Table 3). This corresponds to a seroprevalence of 2.3% and thus to the seroprevalence in the general population of SARS-CoV-2 in Germany at that time [12, 13] and worldwide [12]. Six months later, one more person showed antibodies associated with a SARS-CoV-2 infection (Fig. 1). In this case, the individual was aware that he or she had become infected with SARS-CoV-2, with the history indicating that the infection occurred in the home environment. This is consistent with the results of our earlier study [13], in which 25 employers of the ILM were tested twice for SARS-CoV-2, including physicians, technical assistants, and students. Here again, only one positive incidental finding was found, consistent with the general seroprevalence and an assumed route of infection in the home environmental setting. Furthermore, it is consistent with the rate of postmortem incidental detection of RT-qPCR SARS-CoV-2-positive decedents in 2.4% of all cases [14] and correspondents to a seroprevalence of SARS-CoV-2 between 1.8 and 4.7% of 872 hospital employees of the University Medical Center Hamburg-Eppendorf, Germany [15, 16]. The seroprevalence of SARS-CoV-2 infection-specific antibodies was about the same in recovered COVID-19 patients among in residents of the city of Hamburg (4.2%) in June 2021 [16].

Interestingly, compared to our study, funeral and crematory workers tested for SARS-CoV-2 infection in Qatar in September 2020 had a prevalence of 25.0% and 34.4%, respectively, in a total population of 2.8 million [17] and a general seroprevalence of 13.3% at that time [18]. Even though this prevalence is clearly above ours, this study also assumes that the main source of infection was in the private environment of the participating persons [17]. Otherwise, we could not exclude other and closer physical contacts in Arabic countries due to typical religious measures (e.g., washing).

SARS-CoV-2 infections are normally transmitted by droplet infection and aerosols [19]. However, transmission of the virus via surfaces is possible in principle [20], even though we could not detect viable viruses on surfaces and skin of decedents previously [13, 21, 22]. This is consistent with the findings of Alishaq et al. [17], who additionally found no contamination of the environment of cemeteries and mortuaries with SARS-CoV-2. Due to the still unclear risk of infection when handling COVID-19 decedents in December 2020, many of our participants expressed the suspicion that they might have already undergone a SARS-CoV-2 infection unnoticed. Even though, in our study, 25.0% did not consistently adhere to protective measures in the occupational environment (Table 1), there was no increased seroprevalence.

It should be required that the licensing and practice of funeral professionals be governed by guidelines similar to those used in the health care industry. To date, in Germany, the funeral profession is not protected by law and there are no binding standards [11]. German burial legislation asks for mandatory identification in cases where the decedent poses a risk of infection relevant to third parties. Therefore, concern has arisen not only among physicians who perform autopsies, but also among funeral industry workers who perform external postmortem measures, which could also be associated with a risk of infection. As funeral and crematory workers are not official medical personnel [11], there was no available information, recommendations, or protective measures or adequate equipment for this professional group during the early COVID-19 pandemic and later on. Our study shows that this has led to uncertainty and inconsistent actions within this professional group. In addition, the analysis of the questionnaires revealed that many morticians would like more comprehensive information on infection routes, pathogens, and protective measures. Overall, most felt only partially protected or not protected at all in December 2020 (59.1%), a peak time within the second wave of the worldwide pandemic. Even 6 months later, in June 2021, more than half of the participants (51.6%) still felt partially or not protected (Table 2).

In December 2020, none of the participants were routinely tested for SARS-CoV-2 by their employer. Nevertheless, many participants stated that a test would make them feel safer. Six months later, some participants were tested since April, but interestingly, this did not improve their sense of safety. Rather, it was proposed to work in permanently assigned teams and to pay more attention to standardization of protective measures, due to great variation in the use and handling of protective equipment by the participants. Due to the uncertainty as well as the different measures, external reviews of the protective measures were therefore desired.

In our study, about 50.0% of the participants had received at least one vaccination in June 2021. At that time, a rigid vaccination prioritization applied in Germany [10]. However, funeral and crematory workers were not initially considered to be vaccinated with preference. At the time of the survey, therefore, only a few employees who belonged to prioritization groups had been vaccinated early, as only a few were over 60 years old or had corresponding pre-existing conditions. Interestingly, one company organized the possibility of vaccination for its employees on its own initiative. Other firms tried to interact with the ILM to organize vaccination days. This resulted in the younger workers being vaccinated from April 2021 onwards, although it would not yet have been their turn according to the priority list in Germany. Across Hamburg, 984,009 (53.1%) people had been vaccinated at least once and 663,389 (35.8%) people had been fully vaccinated as of 30 June 2021 and 73.8% by the end of November 2021 [23]. This means that the vaccination rate in our survey was about as high as in the entire city state, even though the workers of the funeral industry had to deal with many COVID-19 deceased on a daily basis. According to their own estimated data, the average number of contacts (including multiple contacts with the same deceased person) with deceased COVID-19 patients among the 33 participants since the beginning of the pandemic averaged 363 per participant.

This assumption shows the burden on funeral and crematory workers due to unclear infection risk and unclear hygiene regulations and protective measures. Already, another study suggests that funeral and crematory workers are generally at higher risk of infection (not only related to the COVID-19 pandemic) and are not adequately trained in protective measures [4]. The risk of SARS-CoV-2 infection in this occupational group apparently corresponds to that of the general population. Nevertheless, a relevant influence of occupational contacts during working hours on the risk of transmission cannot be completely excluded.

## Limitations

During the evaluation, it was noticed that, in many cases, the same answer combinations were recorded, especially in the free text answers. It could not be ensured definitively that multiple entries were made by the same person within one study period. Joint processing of the questionnaires by the participants cannot be ruled out as this was done without supervision. The low numbers of participants in our pilot study are relative for generalizations.

## Conclusion

In our cohort, we found no evidence of an increased risk of transmission of SARS-CoV-2 to workers of the funeral industry. The greatest risk for funeral workers to become infected with the SARS-CoV-2 virus likely comes from unprotected personal contact with the relatives by droplet infections and in the occupational setting. There is a need to provide a better understanding of potentially transmissible infectious diseases with associated protective measures to funeral and crematory workers.

As further—possibly also significantly more contagious—infectious diseases may occur in the future or already exist in other countries, it is necessary to reconsider the classification of the profession of funeral and crematory workers in the health professions with corresponding deeper medical training, especially with regard to infection control measures.

## Key points

1. The funeral industry is an important profession during the corona crisis.
2. Funeral workers in Hamburg may not be at increased risk of contracting SARS-CoV-2.
3. SARS-CoV-2 seroprevalence in funeral workers and the Hamburg population is similar.
4. Education about communicable diseases in the funeral industry should be implemented.

## Data Availability

All data supporting the findings of this study are available within the paper and its Supplementary Information.
